# Knee-ultrasound: reference values for the amount of joint fluid and synovial appearances in healthy children and adolescents

**DOI:** 10.1007/s00247-025-06243-0

**Published:** 2025-05-02

**Authors:** Sílvia Costa Dias, Diogo Costa Carvalho, Miguel Castro, Cláudia Camila Dias, Isabel Ramos, Iva Brito, Karen Rosendahl

**Affiliations:** 1 Faculty of Medicine of the University of Porto, Rua Pedro Hispano nº 190, 2º Dto, Porto, 4100-393 Portugal; 2 Radiology Department, University Hospital Center of São João Porto, Porto, Portugal; 3 Knowledge Management Unit, Faculty of Medicine of the University of Porto, Porto, Portugal; 4 CINTESIS@RISE, Department of Community Medicine, Information and Health Decision Sciences (MEDCIDS), Faculty of Medicine of the University of Porto, Porto, Portugal; 5 Paediatric and Young Adult Rheumatology Unit, University Hospital Center of São João, Porto, Portugal; 6 Department of Clinical Medicine, Faculty of Health Sciences, UiT the Arctic University of Norway, Tromsø, Norway; 7 Section of Paediatric Radiology, University Hospital of North Norway, Tromsø, Norway

**Keywords:** Knee, Musculoskeletal ultrasound, Suprapatellar recess, Synovial fluid, Synovial membrane

## Abstract

**Background:**

With increasing ultrasound use, data for the normal amount of joint fluid and synovial appearances are needed.

**Objective:**

To establish reference values for knee-ultrasound in healthy volunteers by age. To examine the association of joint fluid and synovial appearances with age, sex, and time spent in sports activities.

**Materials and methods:**

Prospective, cross-sectional, including 3–17-year-old volunteers. Examinations were performed by one of two experienced radiologists after meticulous standardisation of the technique. Knees were examined in 30° flexion, in extension and during provocation.

**Results:**

One hundred twenty-seven volunteers (67 females), median age 10.9 years (percentile 2.5^th^-97.5^th^: 3.4–17.2), were included. With 30° knee flexion, the median amount of suprapatellar recess joint fluid (sagittal view) was 0.5 mm for females and 0.9 mm for males (*P* = 0.014), increasing to 1.9 mm and 2.4 mm (*P* = 0.031), respectively, during provocation. The amount of fluid during provocation increased with age for both sexes (*P* = 0.007 females; *P* = 0.012 males) and by time spent in sport-activities (*P* = 0.003). Further, there was a positive association between the presence of joint fluid speckles and sport-activities (*P* = 0.039). Median double-layered synovium was 1.5 mm in 3–6-year-olds, increasing to 2.1 mm in 7–9-year-olds and 2.0 mm in 10–17-year-olds (*P* = 0.024). No synovial Doppler signal was seen. Observers’ agreement was excellent, with ICC values ≥ 0.9.

**Conclusion:**

The majority of healthy children have measurable knee joint fluid, more so when examined with a flexed knee and under provocation. Speckles within the joint fluid are seen in one-fourth, especially in males, and the presence of speckles and joint fluid amount are associated with sports-activity.

**Graphical Abstract:**

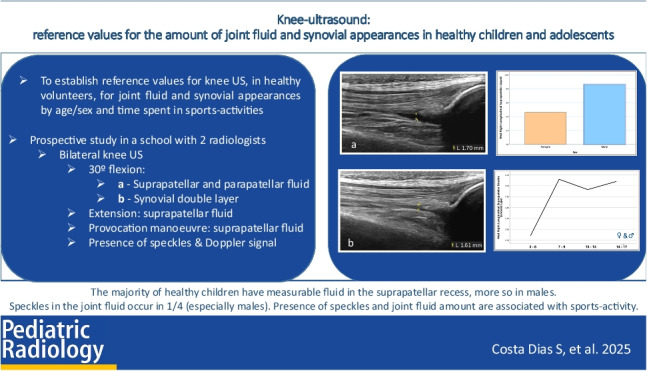

**Supplementary Information:**

The online version contains supplementary material available at 10.1007/s00247-025-06243-0.

## Background

Recent advancements in ultrasound imaging technology have provided exquisite high-resolution images allowing for detailed differentiation between synovium and joint fluid, with the potential to improve diagnosis and follow-up of juvenile idiopathic arthritis (JIA). JIA is characterised by chronic synovial inflammation, e.g. thickened, hyperaemic synovium and effusion, with a potential risk of developing progressive joint destruction and serious functional disability [1, 2]. Recent research has shown that adult disease is seen in up to 70% of those having suffered JIA, underscoring the importance of close monitoring of disease activity to guide therapy during childhood [3]. Normally, joints contain a small amount of joint fluid; however, current literature on reference values is sparse, with a few studies addressing the wrist, elbow, or hip joints [4–6]. For knees, there are two main studies, one including 60 children aged 2–16 years (2007) [7] and one including 257 children aged 1–18 years (2016) [8]. Both studies have published reference values for the depth of the suprapatellar recess, of which one by age and sex [8]. However, none of the studies has distinguished between synovium and joint fluid, or addressed potential differences in appearances by knee positioning and provocation. Moreover, no studies have mentioned the presence of small echogenic dots, or speckles within normal joint fluid that might be seen in some children.

The purpose of our study is to provide ultrasound-data on the presence, amount, and appearances of joint fluid and the appearances of synovium of the knee joint in healthy children and adolescents, by sex, age, and sport-activities.

## Materials and methods

### Study design

This was a single-centre, prospective, cross-sectional study including volunteers from a private school in Porto/Portugal. The study was approved by the Institutional Ethics Committee (IEC) (registration number CE 191/22) and by the school board. Written, informed consent was obtained from all the participants and caretaker(s). Children aged 3–17 years were invited to participate via invitations sent to parents’ representatives. Exclusion criteria were (a) chronic medicated disease that might affect the skeletal system or (b) having experienced a knee trauma within the last 2 months.

### Questionnaire on diseases and knee pain: leg-length assessment

All participants/caretakers filled in a questionnaire on diseases, medication, knee pain/symptoms, and sport-habits (number of sports and hours per week). Knee pain/symptoms were scored as 0 = never, 1 = sporadic, 2 = sometimes, or 3 = frequent. In addition, leg length discrepancy was assessed subjectively at 0.5-cm intervals, by levelling the iliac crests with the child in an upright position.

### Ultrasound examination of the knees

The ultrasound examinations were performed between October 17 and 21, 2022, by one of two examiners (paediatric and musculoskeletal radiologists with 10/12 years of experience, respectively), following meticulous discussions and standardisation of the ultrasound protocol [7–10]. All examinations were performed on a Logiq E10 machine—Version R3 (V.3.2), General Electric (GE Healthcare, Milwaukee, WI), with a linear 6–15-MHz transducer and defined pre-sets. To compare colour and power Doppler, both techniques were performed in the first 23 participants, whilst the remainder were examined with colour Doppler alone. We used low flow settings (adjusted from Ting et al. [9]), including a pulse repetition frequency < 1.0 (0.6), low wall filter (51 Hz), and frequency adjusted to obtain maximum sensitivity, as well as gain (set just below artefact levels). Signal was only considered if located within the synovial area, and scored as 0 (no signal); 1 (1 to 3 signals); 2 (> 3 signals or confluent signals in < 50% of area of synovium); or 3 (confluent signals in > 50% of area of synovium), according to Ting et al. [9].

**Fig. 1:**
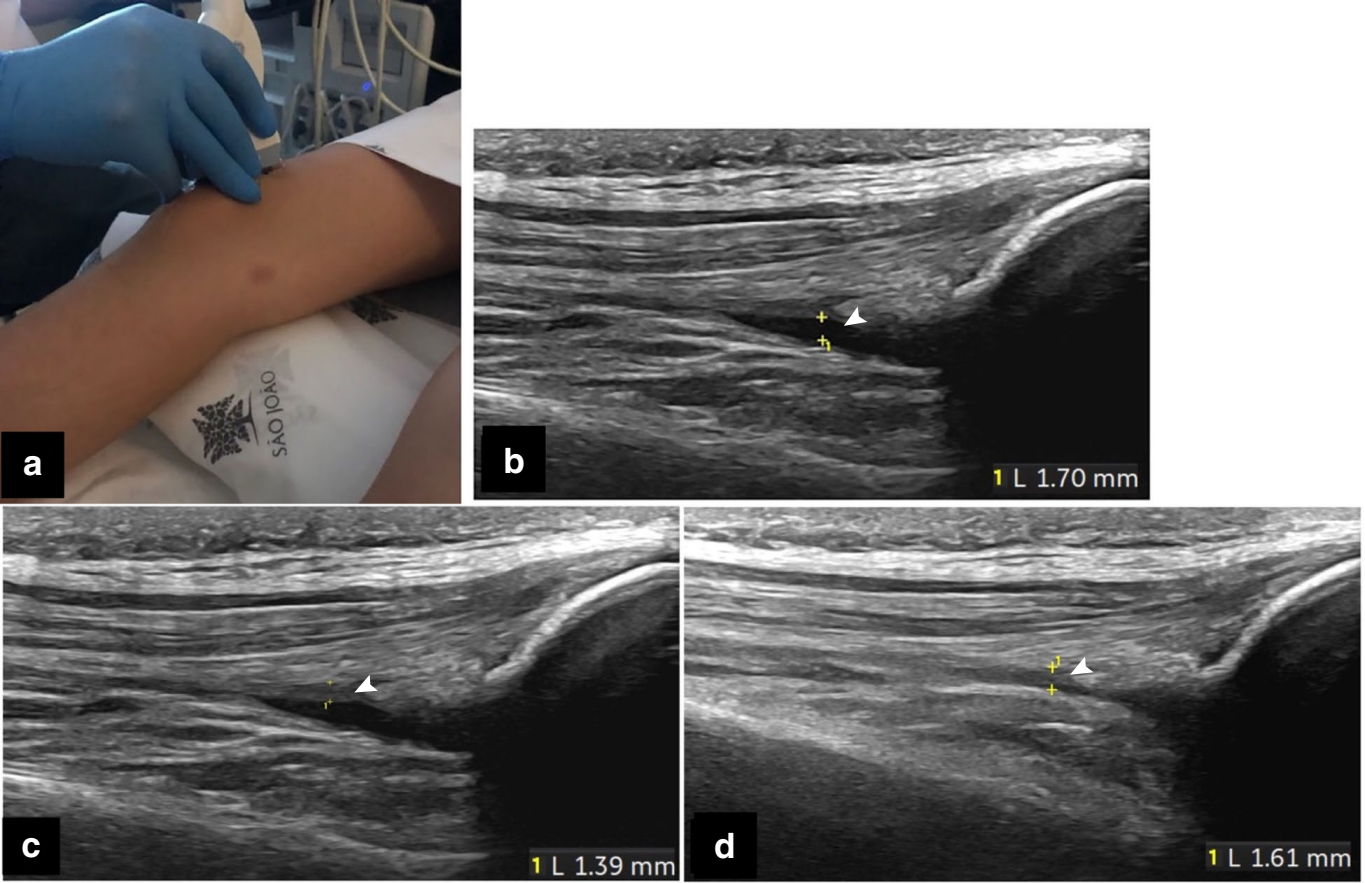
**a** Knee 30° flexed, in a 10-year-old healthy boy, accessing the suprapatellar recess—central longitudinal view (“standard”). **b**-**d** A 13-year-old healthy boy, with 30° knee flexion, central longitudinal standard view. **b** Anteroposterior measurements of fluid (*arrowhead*). **c** Single synovial layer (*arrowhead*). **d** Double synovial layer (during light transducer compression to squeeze fluid away) (*arrowhead*)

**Fig. 2:**
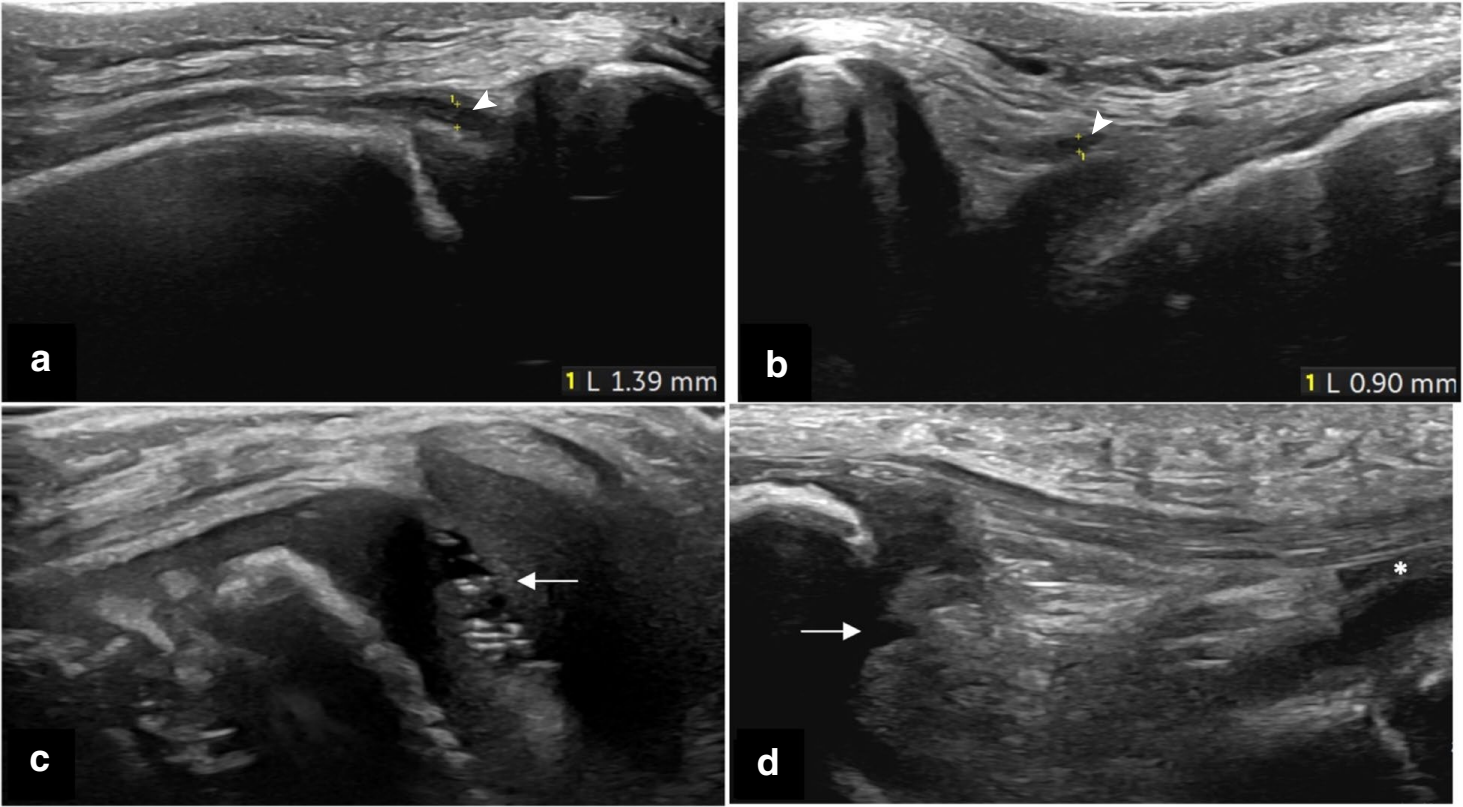
Knee 30° flexed in healthy children. **a**,** b** Transverse views, with measurement of fluid (*arrowheads*) in lateral (**a**) and medial (**b**) parapatellar recesses in a 13-year-old boy. **c** Speckles in the lateral joint space (*arrow*) in a 7-year-old girl. **d** Infrapatellar recess in a 4-year-old girl: fluid is seen underneath the lower pole of the patella (*arrow*) and in the deep infrapatellar bursa (*asterisk*)

**Fig. 3:**
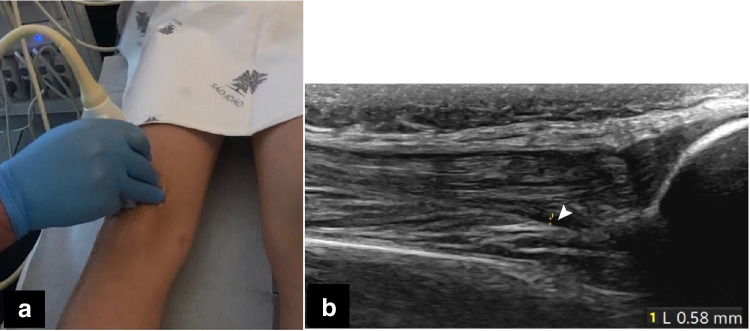
Knee extended in healthy children. **a** Same participant as Fig. 1a, a 10-year-old boy, using the standard longitudinal view. **b** Same participant as Fig. 1b-d, a 13-year-old boy, anteroposterior measurement, showing less fluid in the recess (*arrowhead*)

**Fig. 4:**
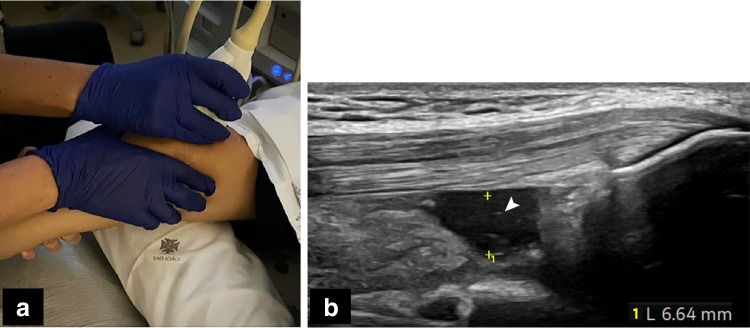
Provocation manoeuvre, knee 30° flexed in healthy children.** a** With one hand squeezing from side to side, whilst performing an upward movement (same participant in Fig. 1a, a 10-year-old boy). **b** Fluid measurement (longitudinal standard view) (*callipers*) in a 16-year-old boy. Note speckles in the fluid (*arrowhead*)

With the participant supine, both knees were examined in 30° flexion, using a longitudinal (standard) view (Fig. 1) and thereafter a transverse view through the lateral and medial parapatellar recesses (Fig. 2). Doppler signals were scored. The presence of hyperechoic, small dots/speckles within the joint fluid was noted for each of the recesses, also including lateral and medial sections, and below the parapatellar view (Fig. 2). Further, the infrapatellar recess was assessed, with fluid measurements underneath the lower pole of the patella and in the deep infrapatellar bursa (Fig. 2). The suprapatellar recess was also examined with knees extended (Fig. 3) and under provocation (Fig. 4).

For inter-observer reproducibility, a second observer, blinded to the first results, examined the right knees in 27 participants within minutes of the first observer.

### Statistical analysis

Except for age and height, continuous variables were not normally distributed; thus, non-parametric tests (Mann–Whitney or Kruskal–Wallis) were applied, using median (Mdn) and percentiles. Age-related values with Mdn 2.5^th^ and 97.5^th^ percentiles were calculated for four age groups (3–6, 7–9, 10–13, and 14–17 years). The Wilcoxon test was used for paired samples. To test the independence of categorical variables, the independent chi-square or the Fisher exact test was applied. The reproducibility between two readers was analysed for 27 right knees, using intra-class correlation coefficient (ICC) with 95% confidence intervals (0–0.39 = poor; 0.40–0.59 = fair, 0.60–0.74 = good, and 0.75–1.00 = excellent agreement) [11] and Bland–Altman mean-difference plots. The mean difference and standard deviation of the differences are reported as measures of bias and variability within or between observers. Twice this standard deviation is defined by the British Standards Institution as a “repeatability coefficient”. For categorical variables, simple Cohen kappa with 95% confidence intervals was used. A *P*-value < 0.05 was considered a priori statistically significant. The reported *P*-values are two tailed. The statistical analysis was performed in Statistical Package for the Social Sciences (SPSS®) v.27.0 and Jamovi (Version 2.3) (computer software).

## Results

A total of 127 participants (67 female) were included. Median age was 10.9 years (PCTL 2.5 ^th^- 97.5^th^ 3.4–17.2 years). Demographic and clinical characteristics of the participants by sex are listed in Table 1.

**Table 1:** Descriptive statistic: study population

Participants	Total	Females	Males	*P*–value^a,b^
*n* (%)	127 (100%)	67 (52.8%)	60 (47.2%)	
Age years, Mdn (percentile 2.5^th^–97.5^th^)	10.9 (3.4–17.2)	10.9 (3.3–16.8)	10.9 (3.3–17.3)	0.723^a^
Height cm, Mdn (percentile2.5^th^–97.5^th^)	143.3 (97.6–182.9)	143.0 (95.1–170.0	143.5 (98.1–186.1)	0.467^a^
Weight kg, Mdn (percentile 2.5^th^–97.5^th^)	37.4 (15.4–72.8)	38.1 (15.6–69.8)	34.9 (14.8–74.3)	0.961^a^
BMI Mdn (percentile 2.5^th^–97.5^th^)	25.5 (15.5–41.6)	26.4 (16.3–43.3)	24.2 (15.0–41.6)	0.927^a^
Race – Caucasian, *n* (%)	123 (96.9)	64 (95.5)	59 (98.3)	0.621^b^
Past medical history, *n* (%)	18 (14.2)	7 (10.4)	11 (18.3)	0.203^b^
Medication^c^, *n* (%)	4 (3.2)	1 (1.5)	3 (5.0)	0.343^b^
Trauma last 2–6 months, *n* (%)	2 (1.6)	2 (3.0)	0 (0.0)	0.498^b^
Previous history OSD (knees) *n*/individuals	5/4	2/2	3/2	
Knee symptoms (mainly mild pain)				
Sporadic or sometimes, *n* (%)	18 (14.2)	13 (10.2)	5 (8.3)	0.074^b^
Sport-activities, *n* (%)				
Number sports, Mdn (percentile 2.5^th^–97.5^th^)	1.0 (0.0–3.0)	1.0 (0.0–3.3)	1.0 (0.0–3.0)	0.932^a^
Total hours/week, Mdn (percentile 2.5^th^–97.5^th^)	3.0 (0.0–10.0)	2.0 (0.0–12.1)	3.0 (0.0–10.0)	0.080^a^
Limb discrepancy, *n* (%)	16 (12.8)	8 (11.9)	8 (13.3)	0.813^b^
Left limb shorter, *n*	10	4	6	0.608^b^

*BMI* body mass index, *Mdn* median, *OSD* Osgood-Schlatter disease

^*a*^Mann-Whitney

^*b*^Chi-square test

^*c*^Other than chronic anti-inflammatory or immunosuppressant drugs

### Joint fluid

From the 127 participants, 96 (75.6%) had visible joint fluid in at least one of the suprapatellar recesses, knee 30° flexed, whilst 62 (48.8%) had bilateral fluid. Apart from the lateral parapatellar recess (right > left, *P* = 0.004), the amount of joint fluid did not differ according to side; thus, the main results are given for the right side only.

The Mdn amount of suprapatellar fluid for females was 0.5 mm with the knee 30° flexed, vs. 0.9 mm for males (*P* = 0.014), rising to 1.9 mm and 2.4 mm, respectively, under provocation (*P* = 0.031). No such difference was seen in either of the parapatellar views (Table 2). In extension, the Mdn amount of fluid was 0.0 mm for both sexes, and thus significantly lower as compared to 30° flexion (*P* < 0.001).

**Table 2:** Amount of fluid in the recesses in millimetres, median with 2.5^th^–97.5^th^ percentiles for 127 children, the right knee, by location and sex

Location	Female (*n* = 67)	Male (*n* = 60)	*P*–value^a^
Longitudinal suprapatellar			
30° flexion, Mdn (Percentile 2.5^th^–97.5^th^)	**0.5** **(0.0–2.4)**	**0.9** **(0.0–2.6)**	**0.014**
Extension, Mdn (Percentile 2.5^th^–97.5^th^)	0.0 (0.0–0.9)	0.0 (0.0–1.3)	0.239
Provocation, Mdn (Percentile 2.5^th^–97.5^th^)	**1.9** **(0.4–4.5)**	**2.4** **(0.8–5.8)**	**0.031**
Transverse parapatellar			
Lateral, Mdn (Percentile 2.5^th^–97.5^th^)	0.0 (0.0–1.8)	0.0 (0.0–2.3)	0.162
Medial, Mdn (Percentile 2.5^th^–97.5^th^)	0.0 (0.0–1.4)	0.0 (0.0–1.7)	0.843

*Mdn* median

^a^Mann-Whitney test

#### Females

In total, 36/67 (53.7%) had visible fluid in the suprapatellar recess (range 0.5–2.7 mm) whilst this was true for 29 (43.2%) of the medial parapatellar (range 0.3–1.4 mm), and 25 (37.3%) of the lateral parapatellar recesses (range 0.4–1.8 mm). The amount of fluid in the lateral and medial parapatellar recess, as well as the amount of fluid on provocation, increased significantly across age groups (Table 3).

**Table 3:** Amount of fluid in the recesses in millimetres, median with 2.5^th^–97.5^th^ percentiles for 67 females, the right knee, by location and age group

Location	Age category, years				*P*–value^a^
	3–6 (*n* = 10)	7–9 (*n* = 16)	10–13 (*n* = 29)	14–17 (*n* = 12)	
Longitudinal suprapatellar					
30° flexion, Mdn (Percentile 2.5^th^–97.5^th^)	0.5 (0.0–1.3)	0.0 (0.0–2.4)	0.6 (0.0–2.2)	0.3 (0.0–2.0)	0.796
Extension, Mdn (Percentile 2.5^th^–97.5^th^)	0.0 (0.0–1.0)	0.0 (0.0–0.8)	0.0 (0.0–0.8)	0.0 (0.0–0.0)	0.367
Provocation, Mdn (Percentile 2.5^th^–97.5^th^)	**1.1** **(0.2–2.3)**	**1.9** **(0.3–3.3)**	**2.2** **(0.9–4.4)**	**1.8** **(1.0–4.6)**	**0.007**
Transverse parapatellar					
Lateral, Mdn (Percentile 2.5^th^–97.5^th^)	**0.0** **(0.0–0.0)**	**0.0** **(0.0–0.7)**	**0.0** **(0.0–1.8)**	**1.0** **(0.0–1.4)**	** < 0.001**
Medial, Mdn (Percentile 2.5^th^–97.5^th^)	**0.0** **(0.0–0.2)**	**0.0** **(0.0–1.1)**	**0.6** **(0.0–1.1)**	**0.6** **(0.0–1.4)**	**0.027**

*Mdn* median

^a^Kruskal-Wallis test

#### Males

In total, 44/60 (73.3%) had fluid in the suprapatellar recess (range 0.6–3.0 mm), whilst this was true for 23 (38.3%) in the medial (range 0.5–1.8 mm), and 27 (45.0%) in the lateral parapatellar recesses (range 0.5–2.4 mm). The amount of fluid in the lateral parapatellar recess, as well as the amount of fluid on provocation, increased significantly by age (Table 4).

**Table 4:** Amount of fluid in the recesses in millimetres, median with 2.5^th^–97.5^th^ percentiles for 60 males, the right knee, by location and age group

Location	Age category, years				*P*–value^a^
	3–6 (*n* = 10)	7–9 (*n* = 13)	10–13 (*n* = 25)	14–17 (*n* = 12)	
Longitudinal suprapatellar					
30° flexion, Mdn (Percentile 2.5^th^–97.5^th^)	0.8 (0.0–2.4)	0.8 (0.0–1.6)	1.0 (0.0–2.5)	0.8 (0.0–2.7)	0.841
Extension, Mdn (Percentile 2.5^th^–97.5^th^)	0.0 (0.0–0.5)	0.0 (0.0–0.7)	0.0 (0.0–1.0)	0.0 (0.0–1.6)	0.628
** Provocation, Mdn** **(Percentile 2.5**^**th**^**–97.5**^**th**^**)**	**1.9** **(1.0–3.6)**	**1.7** **(0.7–3.2)**	**2.8** **(0.9–5.2)**	**3.1** **(1.5–6.3)**	**0.012**
Transverse parapatellar					
** Lateral Mdn** **(Percentile 2.5**^**th**^**–97.5**^**th**^**)**	**0.0** **(0.0–0.4)**	**0.0** **(0.0–1.2)**	**0.0** **(0.0–2.2)**	**1.1** **(0.0–2.4)**	**0.001**
Medial Mdn (Percentile 2.5^th^–97.5^th^)	0.0 (0.0–0.0)	0.0 (0.0–1.1)	0.0 (0.0–1.8)	0.6 (0.0–1.3)	0.053

*Mdn* median

^a^Kruskal-Wallis test

### Speckles within the joint fluid

Speckles in at least one recess were seen in 26 right knees (20.5%), in 18 (14.2%) of the suprapatellar recess, in 9 (7.1%) of the lateral and in 4 (3.1%) of the medial parapatellar recesses. In 56 (44.1%) right knees, there were speckles along the lateral joint space, whilst speckles along the medial joint space were seen in 30 (23.6%).

The number of recesses containing at least one speckle was lower for females, with at least one positive recess in 6 of the females (9.0%) versus 20 of the males (33.3%) (*P* < 0.001). Considering just the suprapatellar recess, the figures were positive in 3 females (4.5%) versus 15 males (25.0%) (*P* < 0.001).

### Associations between joint fluid, speckles, and additional features

**Fig. 5:**
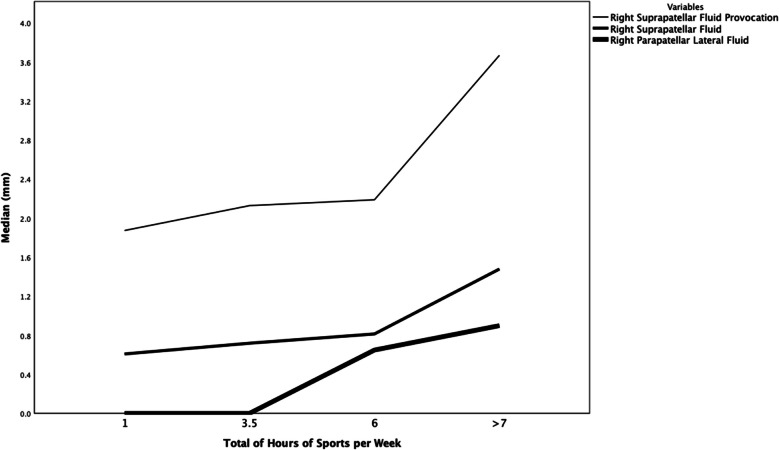
Median amount of joint fluid millimetres in the right suprapatellar recess, with/without provocation, and in the lateral parapatellar recess through total hours of sports activities per week

The amount of joint fluid in the right suprapatellar recess increased significantly by the total hours of weekly sport-activity, both when based on the 30° flexed view (*P* = 0.028) and on provocation (*P* = 0.003), and in the lateral parapatellar recess (*P* = 0.029) (Fig. 5). Similarly, there was a significant association between total hours of sports-activity and the presence of speckles in the joint fluid (*P* = 0.039), with higher Mdn of hours for children with speckles (3.5 h vs. 2.0 h per week for children without speckles). No statistically significant associations were found between the amount of joint fluid and the presence of speckles, with past medical history, symptoms, medication, trauma, or lower limb length discrepancy.

### Fluid in other locations

Fluid underneath the inferior pole of the right patella was identified in 3 (15.0%) children aged 3–6 years and in 2 (6.9%) children aged 7–9 years, but not in the older age groups (*P* = 0.017). Of the 127 participants, 12 (9.4%) had some fluid in the right deep infrapatellar bursa, with no differences according to age (*P* = 0.537). For both findings, there were no differences according to sex (*P* = 0.542 and 0.823) or symptoms (*P* = 0.735 and 0.754).

### Synovial thickness and Doppler signal

For the suprapatellar recess, no significant differences in thickness of the double-layered synovium were seen according to side (*P* = 0.714) or sex (*P* = 0.627). However, significant differences were found according to age groups. Thus, synovial thickness of the double-layered synovium is given for the right knee by age group (Table 5). A simple linear regression based on the right measures showed an equation, in which synovial double-layer thickness is predicted in millimetres based on age, with *P* < 0.001 (Table 5). No significant associations were found between synovial thickness and past medical history, medication, trauma, or leg length discrepancy.

**Table 5:** Synovial thickness in millimetres, median with 2.5^th^–97.5^th^ percentiles in 127 children (60 males), the right knee, by age group. Equation to predict synovial thickness in millimetres based on age in years

Location	Age category, years				*P*–value^a^
	3–6 (*n* = 20)	7–9 (*n* = 29)	10–13 (*n* = 54)	14–17 (*n* = 24)	
Longitudinal suprapatellar					
30° flexion, Mdn (Percentile 2.5^th^–97.5^th^)	**1.5** **(1.1–2.5)**	**2.1** **(1.2–2.9)**	**2.0** **(1.3–3.9)**	**2.0** **(1.4–3.3)**	**0.024**
Synovial thickness (mm) = 1.493 + 0.05 × age in years					

*Mdn* median, *mm* millimetres

^a^Kruskal-Wallis test

No colour Doppler signal was seen within the synovium in either of the ultrasound views performed. In the first 23 children, an additional power Doppler examination was performed. No Doppler signal was seen on either of the two techniques; thus, for the remainder 104 participants, only colour Doppler was obtained.

### Inter-observer agreement

Inter-observer agreement on the assessment of fluid and synovial thickness was excellent, with ICC values ≥ 0.9. One-sample *t*-tests showed that the corresponding mean difference (bias) was not significantly different from zero for fluid measurements or synovial thickness (*P* > 0.999 and *P* = 0.899, respectively). Ninety-five per cent limits of agreement were relatively wide for fluid (± 65% of the sample mean) and fair for synovial thickness (± 35% of the sample mean) (Supplementary Material 1). The agreement for identification of speckles and scoring of Doppler signals was excellent, with kappa values between 0.9–1.0 for speckles and 1.0 for Doppler.

## Discussion

We have presented reference values for the amount of joint fluid and synovial thickness of the knee in healthy children and adolescents, examined for research purposes only. To the best of our knowledge, this is the first study to assess joint fluid and synovium separately, and to describe speckles within the joint fluid. More than 75% of the 127 volunteers had visible joint fluid in at least one of the suprapatellar recesses with the knee 30° flexed, whilst nearly half had bilateral fluid. Of note is that the amount of fluid was higher with the knee 30° flexed as compared to extended, that males had more fluid than females, and that the amount increased with age and on provocation. Moreover, that speckles were seen in at least one of the recesses in one-fourth of the participants, more so in males, and that both the presence of speckles and the amount of joint fluid were associated with sports-activity.

Our results compare in part with those of others, although the examination techniques differ slightly. In a study of 60 healthy children aged 2–16 years-old, Collado et al. [7] found fluid in 60% of the suprapatellar recesses with knees in 30° flexion, as compared to 75% in our cohort. However, they applied pressure with the ultrasound probe whilst assessing the suprapatellar recess, leading to a fluid reduction. Furthermore, they reported fluid in 58% of the medial and 33% of the parapatellar recesses with the knees extended, which potentially also reduces the fluid. Thus, these technical issues may explain part of the differences.

Similarly, in a multicentre study of 435 children aged 1–18 years old, Windschall et al. [8] found that over 64% of the children had visible fluid in the suprapatellar recess, varying from 18% amongst 1–3-year-olds to 80% of 4–6-year-olds. The results were presented by age categories and sex; however, potential statistically significant differences between sexes were not analysed for these features. Their reported reference values for the suprapatellar recess were generally higher than ours, most likely reflecting differences in examination techniques. Whilst we measured fluid and synovial thickness separately in 30° flexion, followed by an extended knee position, their measurements were taken with the knees in a neutral position, measuring both a double-layered synovium and potential fluid. Our results showed that the amount of fluid in the suprapatellar recess differed significantly according to the position of the knee joint, with more fluid seen in 30° flexion compared to extension, highlighting the importance of a standardised examination technique. We could argue that assessing joint fluid and synovial thickness separately might prove more helpful, particularly when dealing with equivocal cases.

In our study, males had more fluid in the suprapatellar recess, both with the knee slightly flexed and during provocation, as compared to females. No such difference was seen for the parapatellar recesses. Further, no differences were seen according to age in either sex, except for most parapatellar recesses, showing an increase of fluid with age, particularly in the oldest age group and during provocation. The latter is interesting, as it might reflect differences in fluid localisation by age, similar to what has been reported for neonates with septic coxitis [12].

Not unexpectedly, the amount of joint fluid doubled or even tripled during the provocation manoeuvre, underscoring the importance of a standardised technique when monitoring patients with arthritis. We believe that the manoeuvre, mirroring the clinical “patella dip” test, helps understand the bigger picture as compared to one standard view alone. Moreover, the synovium was easier to assess with fluid in the recess.

Interestingly, we found a positive correlation between the amount of joint fluid and hours spent on sport-activities, indicating that mechanical stress might play a role. To our knowledge, this is a novel observation in children. Similarly, knee-MRI after exercise in adults has shown an increase of joint fluid [13, 14].

Fluid underneath the inferior pole of the patella was identified in almost 4% of the children, all under the age of 9. An explanation might be that a less ossified patella provides a better acoustic window. Another interesting finding was that 10% of children had some fluid within the deep infrapatellar bursa, with no differences according to age. Both features should be acknowledged as normal variations and not be mistaken for pathology.

We have previously noted that some children have numerous hyperechoic dots in otherwise anechoic joint fluid, not representing intra-articular gas. We have termed these findings “speckles”. Interestingly, 25% of all the participants had such speckles in at least one recess, more so in males and on the lateral side of the joint space. Moreover, there was an association between the presence of speckles and hours spent on sport-activities. Their clinical significance is unclear, but we speculate that they might be related to joint fluid viscosity or with some fluid components. A study in adult patients with knee osteoarthritis reported a significant increase in joint fluid viscosity and molecular weight of hyaluronan after 12 weeks of exercise [15]; however, there was no ultrasound fluid assessment.

Traditionally, synovial hypertrophy in children has been graded subjectively (mild/moderate/marked). Direct measurements are less commonly used, in part due to challenges with the precision of small measurements. Roth J. et al. [16] stated that synovitis can be detected using B-mode alone, as an abnormal, intra-articular, anechoic, non-displaceable material. In our study, when the double-layered synovium was measured in the suprapatellar recess, exerting a mild pressure to remove fluid, we found a median thickness of around 2 mm, increasing with age. This is intriguing, as the Juvenile Arthritis MRI Scoring (JAMRIS) system [17] has defined synovial hypertrophy as enhancing synovium thicker than 2 mm. Their cut-off between normality and pathology is supported by an MRI-study of healthy children reporting that the normal, enhancing synovium does not exceed 1.8 mm in thickness [18]. Direct comparison between ultrasound and MRI is difficult due to different image technologies; however, ultrasound images have better spatial resolution as compared to MRI.

Measurements of single synovial layer revealed non-significant results. This might be due to difficulties in measuring a single layer, especially in the absence of joint fluid. Of note is that on MRI, measuring synovial thickness in locations without fluid will correspond to our double-layer measurement.

According to Roth et al. [16], both colour and power Doppler perform well in the assessment of hyperaemia. We found no Doppler signal in either of the synovial recesses, suggesting that when present, it indicates pathology. Our findings compare well with others. In a paper by Miotto e Silva et al. [19], the authors found no power Doppler signal in the synovium of 1,224 joints examined, except for one wrist joint in one of the controls. Similarly, Collado et al. [7] found no positive Doppler signal in his study of the knee joints in 60 healthy children.

**Fig. 6:**
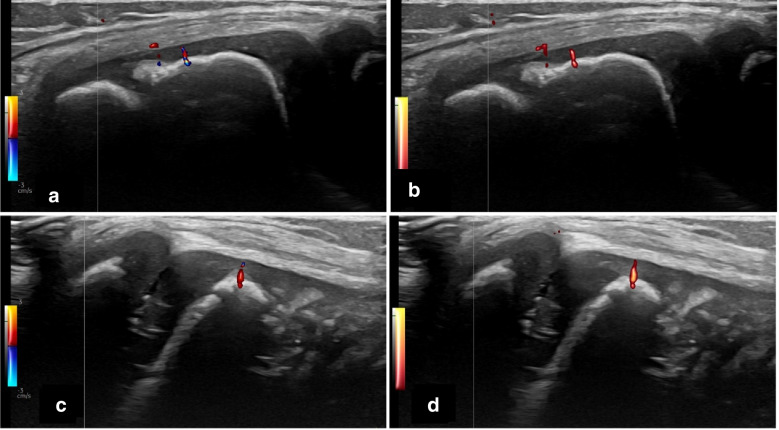
Transverse knee-ultrasound images in two 7-year-old healthy girls, at the level of the lateral parapatellar recess (**a**, **b**) and at the level of the lateral joint space (**c**, **d**). The colour Doppler (**a**,** c**) and power Doppler (**b**, **d**) images show identical findings of normal vessels in cartilage

Our small pilot, comparing the two Doppler techniques, yielded no differences; however, the comparison was hampered by lack of positive cases. Some physiological Doppler signals were seen, like vessels, for example across cartilage (Fig. 6) and in Hoffa’s fat pad.

The strengths of our study are the prospective design, including healthy children examined for research purposes only, and second, the meticulous and extensive ultrasound examinations, using state-of-the art ultrasound equipment. We acknowledge, however, several limitations to our study. First, all participants were recruited from the same school, most being Caucasians, potentially limiting the generalizability. Second, the number of examinations per age group by sex varied, with smaller sample sizes for some of the analysis. Third, although there was an excellent agreement on measuring fluid and synovial thickness based on the ICCs, the reproducibility as assessed by the Bland–Altman approach was only fair, a finding also reported by others [20].

## Conclusion

We have presented novel, ultrasound-based references by age for synovial thickness and fluid in the knee joint, the latter for males and females separately.

## Supplementary Information

Below is the link to the electronic supplementary material.

Supplementary file1 (DOCX 231 KB)

## Data Availability

No datasets were generated or analysed during the current study.
