# Supplementary material for: Knee-ultrasound: reference values for the amount of joint fluid and synovial appearances in healthy children and adolescents
Source: Pediatr Radiol. 2025 May 2;55(6):1127–37. doi: 10.1007/s00247-025-06243-0 (PMC12119740; doi:10.1007/s00247-025-06243-0)
Supplement: Supplementary file 1 — Supplementary file1 (DOCX 231 KB) [file 247_2025_6243_MOESM1_ESM.docx]

Supplementary Material 1

Bland-Altman mean-difference plots in millimetres **a** Showing relative wide 95% limitis of agreement for both the suprapatellar fluid (including repeted values represented by the same dot) and **b** The thickness of the double layered synovium between observers (based on the longitudinal standard view)


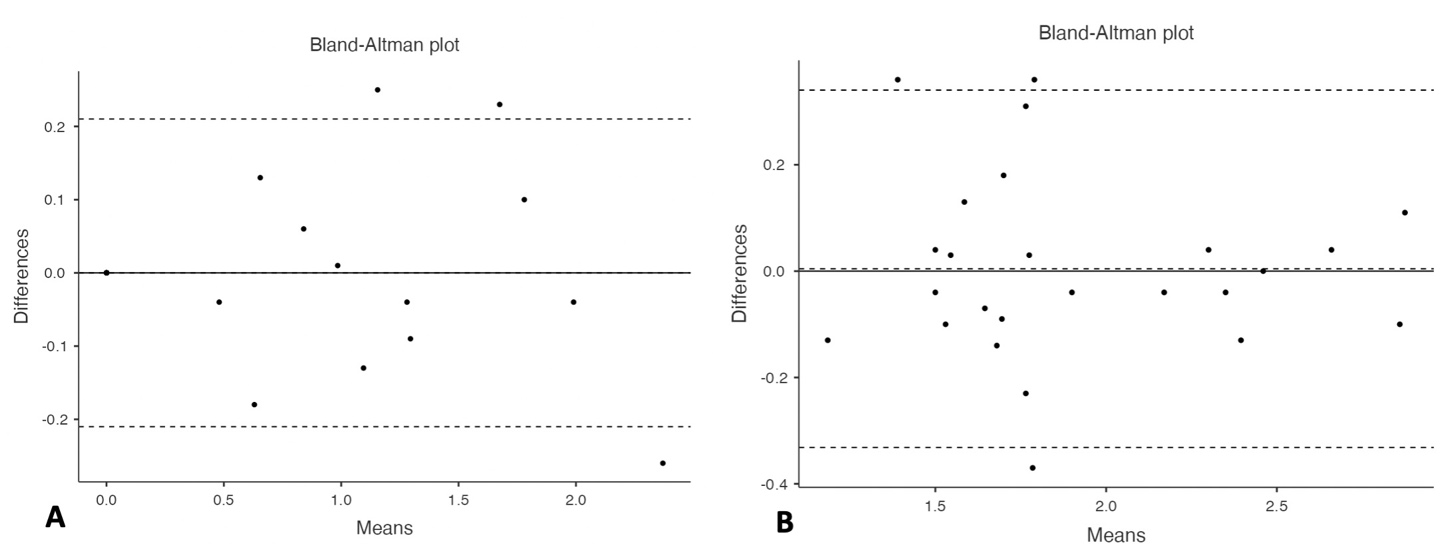


(mm)

(mm)

(mm)

(mm)

**a**

**b**
